# Dynamic Mode Decomposition of Fast Pressure Sensitive Paint Data

**DOI:** 10.3390/s16060862

**Published:** 2016-06-11

**Authors:** Mohd Y. Ali, Anshuman Pandey, James W. Gregory

**Affiliations:** Aerospace Research Center, The Ohio State University, 2300 West Case Road, Columbus, OH 43235, USA; ali.511@osu.edu (M.Y.A.); pandey.46@osu.edu (A.P.)

**Keywords:** pressure-sensitive paint (PSP), polymer/ceramic (PC-PSP), acoustic resonance cavity, dynamic mode decomposition (DMD), proper orthogonal decomposition (POD)

## Abstract

Fast-response pressure sensitive paint (PSP) is used in this work to measure and analyze the acoustic pressure field in a rectangular cavity. The high spatial resolution and fast frequency response of PSP effectively captures the spatial and temporal detail of surface pressure resulting in the acoustic pressure field. In this work, a high-speed camera is used to generate a continuous time record of the acoustic pressure fluctuations with PSP. Since the level of the acoustic pressure is near the resolution limit of the sensor system, advanced analysis techniques are used to extract the spatial modes of the pressure field. Both dynamic mode decomposition (DMD) and proper orthogonal decomposition (POD) are compared with phase averaging for data analysis. While all three techniques effectively extract the pressure field and reduce the impact of sensor noise, DMD and POD are more robust techniques that can be applied to aperiodic or multi-frequency signals. Furthermore, DMD is better than POD at suppressing noise in particular regions of the spectrum and at effectively separating spectral energy when multiple acoustic excitation frequencies are present.

## 1. Introduction

Fast pressure-sensitive paint (PSP) is a class of optical sensors developed in recent years for quantitative measurement of unsteady surface pressure distribution on aerodynamic bodies. It is based on the principles of conventional PSP [1,2] and uses optimized physical structure for improved permeability of the quenching gas. With a frequency response of several kilohertz, Fast PSPs are now capable of resolving highly unsteady flows such as shock-wave fluctuations, transonic buffeting, and surge in turbomachinery [3,4,5]. Furthermore, due to their ability to provide much higher resolution pressure information than surface pressure taps, fast PSPs have found applications in determination of time-resolved loads, validation of unsteady simulations, and understanding the underlying flow physics [4].

**Figure 1:**
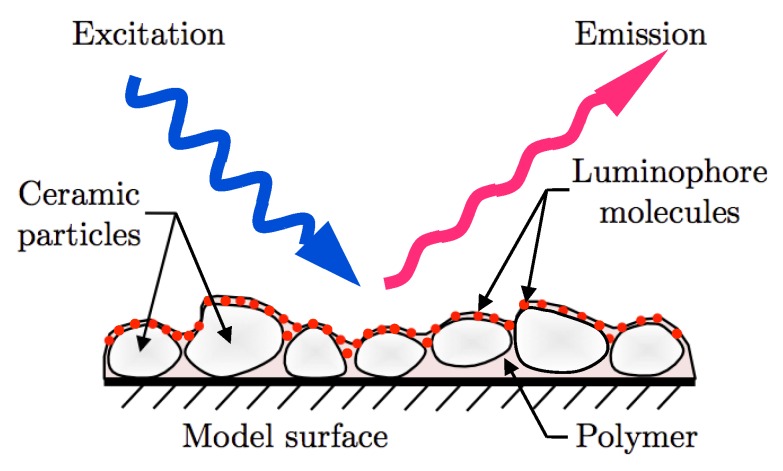
Schematic of Polymer/Ceramic Pressure-Sensitive Paint (PC-PSP).

Pressure sensitive paint is based on the quenching of excited state luminescent molecules (luminophores) by ambient oxygen (Figure 1). These molecules are first brought to the first triplet state through excitation at a particular wavelength and are then allowed to relax back to their ground state through either emission at a stokes-shifted wavelength or by interaction with surrounding oxygen molecules (oxygen quenching). This modulation of light emission by oxygen quenching is the basis for obtaining the partial pressure of oxygen, and, by Henry’s Law, pressure. Fast PSPs use a porous structure for hosting the luminophores, which enables rapid quenching by time-varying changes in pressure. However, since most of the luminophores are already quenched under ambient conditions due to the higher porosity of the binder, fast PSPs have an inherently lower sensitivity than conventional PSPs. Due to this lower sensitivity, fast PSPs have seen limited application in measurement of low-speed unsteady flows that exhibit very small surface pressure fluctuations. Error sources such as temperature sensitivity and shot noise corrupt the fractional changes in PSP emissions [6]. Further reduction in signal-to-noise ratio (SNR) occurs when short-exposure high-speed imaging is used for time-resolved measurements. To counter these error sources and to improve SNR in low-speed flows, phase-averaging methods have traditionally been used for unsteady measurements. The technique requires a consistent periodic pressure signal that is characteristic of the flow feature of interest. A reference transducer signal is typically used to either phase-lock the excitation light (fast-LEDs) or camera exposure for averaging on-board the camera [7,8]. With high-speed cameras, simultaneous recording of camera exposure time-stamps and a reference transducer signal can be used for post-process conditional averaging of the images [8,9]. The disadvantage of this technique is the need for a periodic single-frequency signal for phase-locking the acquisition. Most flow phenomena, however, contain multiple frequencies, thereby precluding the effective use of this technique.

In order to capture broadband flow phenomena, frequency domain binning of PSP data based on the Fast Fourier Transform (FFT) was introduced by Nakakita [10]. An FFT was performed on every pixel of time-resolved images acquired from a high-speed camera to obtain the amplitude spectra. These amplitude spectra were then restructured as camera view images and binning was performed over the frequency domain to extract flow structures at different frequencies. The drawback associated with FFT based methods is the large size of the data record required to obtain a sufficient average. This may introduce errors such as temperature changes or photodegradation of the PSP. Proper orthogonal decomposition (POD) was introduced for noise suppression in non-periodic low-speed PSP measurements by Pastuhoff *et al.* [11]. Unlike phase-averaging, which requires periodicity, this method requires only that the pressure features are uncorrelated with the noise and thus would work on non-periodic cases as well. POD, also known as singular value decomposition (SVD), decomposes the measurements into spatially orthogonal modes, the combination of which provides the most optimal (in the least squares sense) low dimensional reconstruction of the observed phenomena. The spatial eigenfunction of a mode can be used to identify the flow structures, while the temporal coefficient provides its evolution information. This method has been used to identify flow structures on a turret by Gordeyev *et al.* [12] and for quantifying the vortex shedding behind a cylinder by Peng *et al.* [13]. The major drawback of POD is that the method is based on second order flow statistics, thereby limiting the information that can be gained about the physical phenomena. Furthermore, POD ranks the coherent structures according to their energy content. For flows with multiple dominant structures, the selection of modes can become challenging based on energy content.

Dynamic mode decomposition is being increasingly used for fluid flow problems to extract dominant flow features. While DMD has been typically applied to velocity field data (numerical or experimental), application to PSP pressure data has not yet been demonstrated. The objective of the present work is to use dynamic mode decomposition (DMD) to extract the key features of a two-dimensional low-amplitude pressure field. A comparison with the theoretical model of the pressure field and data reduction by phase-averaging, and proper orthogonal decomposition (POD), is also carried out. Experiments are conducted on an acoustic resonance cavity with single or multiple high-frequency acoustic excitation inputs. High-speed imaging is used to capture the PSP response to the pressure fluctuations resulting from the acoustic field.

## 2. Background

### 2.1. Dynamic Mode Decomposition

Dynamic mode decomposition (DMD) is related to the Koopman modes obtained by the classical Arnoldi algorithm [14]. The temporal DMD algorithm used in the present work was adapted from Schmid [15]. The data set to be analyzed is a time-resolved record (snapshot sequence) sampled at a frequency ($f_{s}$) sufficiently high to satisfy the Nyquist criterion. A temporal sequence of *N* snapshots, consisting of column vectors, $v_{j}$, that are equispaced in time, can be written as:

(1) $$V_{1}^{N}=\left\{v_{1},\;v_{2},\;v_{3},\;\cdots ,\;v_{N}\right\}$$

The primary basis of the method is that each snapshot in time, $v_{j}$, is connected to a subsequent snapshot, $v_{j+1}$, by a linear mapping A, such that

(2) $$v_{j+1}=A\;v_{j}$$

where A is constant over the full data set. The eigenvalues and eigenvectors of the matrix A characterize the behavior of the dynamical system. The assumption of constant mapping of the dynamical system, A, between the snapshot sequence allows us to formulate a Krylov sequence of the data,

(3) $$V_{1}^{N}=\left\{v_{1},\;A\;v_{1},\;A^{2}\;v_{1},\cdots ,\;A^{N-1}\;v_{1}\right\}$$

As the number of snapshots increase, the data set is assumed to approach a linear dependency. The last snapshot vector, $v_{N}$, can be expressed as a linear combination of the previous linearly independent vectors,

(4) $$v_{N}=a_{1}\;v_{1}+a_{2}\;v_{2}+\cdots +a_{N-1}\;v_{N-1}+r$$

where r is the residual vector. Equation (4) can be written in a matrix form,

(5) $$v_{N}=v_{1}^{N-1}\;a+r$$

where the coefficients $a^{T}=\left\{a_{1},\;a_{2},\;\cdots a_{N-1}\right\}$ can be obtained using the least squares method. Following Ruhe [16], Equation (5) can be written in the form of two lagged matrices

(6) $$\begin{array}{cc} A\{v_{1},\;v_{2},\;v_{3},\;\cdots ,\;v_{N-1}\} & =\{v_{2},\;v_{3},\;v_{3},\;\cdots ,\;v_{N}\}\\ & =\left\{v_{2},\;v_{3},\;v_{4},\;\cdots ,\;V_{1}^{N-1}\;a\right\}+r\;e_{N-1}^{T} \end{array}$$

where $e_{N-1}$ is the ${\left(N-1\right)}^{\text{th}}$ unit vector. Equation (6) can be written in a matrix form as follows:

(7) $$AV_{1}^{N-1}=V_{2}^{N}=V_{1}^{N-1}S+r\;e_{N-1}^{T}$$

The matrix S in Equation (7) is of the companion type,

(8) $$S=\left(\begin{array}{ccccc} 0 & & & & a_{1}\\ 1 & 0 & & & a_{2}\\ & \ddots & \ddots & & \vdots \\ & & 1 & 0 & a_{N-2}\\ & & & 1 & a_{N-1} \end{array}\right)$$

which shifts the dataset (snapshot sequence) index from one to N-1. The number of snapshots, *N*, can be increased until the residual, r, converges. The matrix S is a low-dimensional representation of the the full system matrix A. The eigenvalues ($\lambda_{j}$) of matrix S approximate some of the eigenvalues of the full system matrix A [15], and are also referred to as the Ritz values [14].

The companion matrix S is computed by calculating the singular value decomposition of the snapshot matrix $V_{1}^{N-1}$,

(9) $$V_{1}^{N-1}=U\;\Sigma \;W^{H}$$

Substituting Equation (9) into Equation (7) and neglecting the residual term, we get

(10) $$V_{2}^{N}=U\;\Sigma \;W^{H}\;S$$

and the approximate ‘full’ matrix,

(11) $$\tilde{S}=U^{H}\;V_{2}^{N}W\;\Sigma^{-1}=U^{H}\;A\;U$$

which is obtained by projecting A on to U. The matrix U contains the POD basis and forms the right singular vector of the snapshot matrix $V_{1}^{N-1}$. The eigenvalue decomposition of the matrix $\tilde{S}$ gives the eigenvalues, $\lambda_{j}$, and eigenvectors, $y_{j}$, such that $\tilde{S}\;y_{j}=\lambda_{j}\;y_{j}$. Finally, the dynamic modes are computed as:

(12) $$\Phi_{j}=U\;y_{j}$$

The approximate eigenvalues (Ritz values), $\lambda_{j}$, can be used to study the stability characteristics of the computed DMD modes. The eigenvalues occur as complex conjugate pairs and lie on a unit circle in the complex domain representing the modes with zero growth rates. The eigenvalues lying inside and outside the unit circle represent the damped and undamped modes, respectively. Furthermore, the eigenvalues can be mapped logarithmically as

(13) $$\omega_{j}=\log\left(\lambda_{j}\right)/\Delta t$$

where $\Delta t=f_{s}^{-1}$ is the separation time between successive snapshots. The discrete frequencies of the decomposed data, $f_{j}$, are determined from the imaginary part of the logarithmically mapped eigenvalues as

(14) fj=2πIm{ωj}=arg(λj)/(2πΔt)

The negative frequencies are neglected and each mode pair is identified by the positive-valued frequency. The mean feature is a special case of DMD with zero eigenvalue (frequency), indicating that it is invariant in time. Beyond the zero-frequency case, the DMD modes can be sorted by their amplitudes, ||Φ||.

Dynamic mode decomposition is closely related to the more commonly used POD modes. The POD modes can also be analyzed from the above discussed analysis. The singular value decomposition of matrix $V_{1}^{N-1}$ (Equation (9)) contains the spatial structures (U, referred to as ‘*topos*’), the eigenvalues (diagonal matrix **Σ**) according to which the POD modes are ranked, and the temporal structures (W, known as ‘*chronos*’). In POD, spatial orthogonality of the identified structures is enforced, leading to multiple frequencies in each individual POD mode. A key difference between POD and DMD is that dynamic mode decomposition aims at an orthogonality in time by identifying pure frequencies.

### 2.2. Acoustic Theory

The theoretical driving frequency *ω* for a particular mode shape in an acoustic resonance cavity is obtained from a solution of the Helmholtz equation [17] and is given by,

(15) $$\omega =c\;\pi \sqrt{{\left(\frac{n_{x}}{L_{x}}\right)}^{2}+{\left(\frac{n_{y}}{L_{y}}\right)}^{2}+{\left(\frac{n_{z}}{L_{z}}\right)}^{2}}$$

where $L_{x}$, $L_{y}$, and $L_{z}$ are the length, height, and depth in the *x*, *y*, and *z* directions, respectively; $n_{x}$, $n_{y}$, and $n_{z}$ are the mode numbers along the same dimensions; and *c* is the speed of sound. Thus, for a given resonance cavity and air temperature, a particular mode $\left(n_{x},\;n_{y},\;n_{z}\right)$ will have a specific resonance frequency that should be excited. The theoretical pressure distribution is given as [17]

(16) $$P\left(x,y,z\right)=\cos\left(\frac{n_{x}\;\pi \;x}{L_{x}}\right)\cos\left(\frac{n_{y}\;\pi \;y}{L_{y}}\right)\cos\left(\frac{n_{z}\;\pi \;z}{L_{z}}\right)$$

For a given mode shape, the driving frequency of the speaker is tuned such that a maximum pressure is obtained in the transducer signal. This ensures that resonance is achieved in the acoustic box. At a given resonance frequency, the sound pressure level (SPL) is defined in terms of the root-mean-square pressure fluctuation for a sine wave as

(17) $$\text{SPL}=20\log_{10}\left[\frac{P/\sqrt{2}}{P_{\text{ref}}}\right]$$

where *P* is the pressure amplitude from the transducer signal (Pa) and $P_{\text{ref}}=20\times 10^{-6}$ Pa is the reference pressure.

## 3. Experimental Framework

### 3.1. Apparatus and Instrumentation

**Figure 2:**
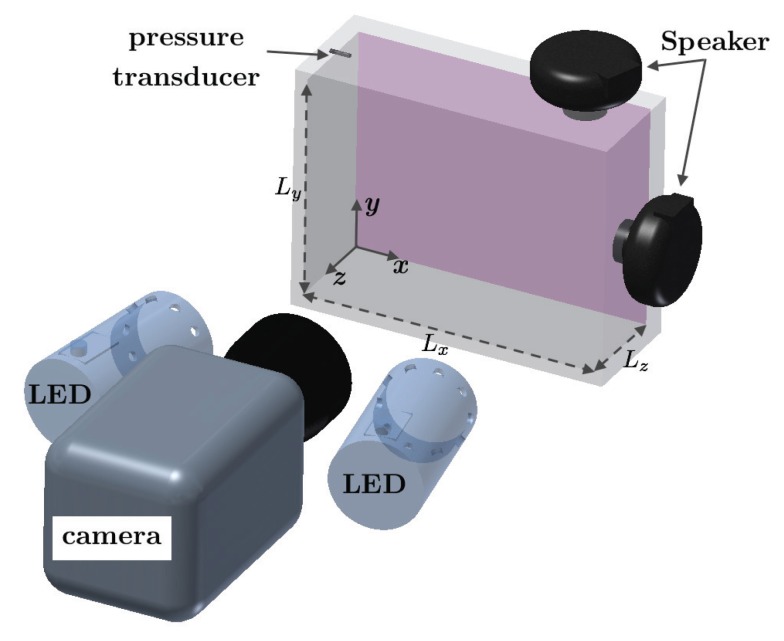
Acoustic box with instrumentation.

The acoustic resonance cavity and instrumentation used in this work are depicted in Figure 2. The inner dimensions of the acoustic box were, length $L_{x}=216$ mm, height $L_{y}=168$ mm, and depth $L_{z}=102$ mm. The cavity walls were made of acrylic with a thickness of 12.7 mm. The box was secured to the optics table using clamps and rubber straps to minimize vibrations. Two acoustic speakers (Atlas Sound Compression Speakers—AS100N) with maximum output of 100 W each were mounted on the top ($x/L_{x}=0.9,\;y/L_{y}=1,\;z/L_{z}=0.375$) and side ($x/L_{x}=1,\;y/L_{y}=0.5,\;z/L_{z}=0.5$) walls of the box via 19-mm diameter holes. The speakers were configured to propagate sound waves into the box and generate various mode shapes. Experiments were conducted with either one or two speakers present, depending on whether simultaneous frequencies were excited. The speakers were driven by two function generators (Agilent 33220A, Englewood, CO, USA) connected to a dual-channel amplifier (Crown XLS 1000, Elkhart, IN, USA). The acoustic box was also instrumented with an Endevco pressure transducer (model 8507C-1), mounted in a corner of the box such that the two-dimensionality of the excited spatial modes can be exploited. The pressure transducer was powered using a signal-conditioner, with a linear gain of 100 applied to the transducer signal. The transducer signal was recorded on an oscilloscope (LeCroy WaveRunner 44Xi, Chestnut Ridge, NY, USA).

A high-speed, 12-bit CMOS camera (Vision Research Phantom V1210, Oakbrook Terrace, IL, USA) with a maximum frame rate of 12,600 fps at full resolution (1280×800 pixels) was used to record the light emission from the PSP. A Nikon 55 mm lens with a 590-nm colored long pass-filter (Schott OG590, Barrington, NJ, USA) was used to separate the excitation light from the paint emission. The present measurements were recorded at a resolution of 640×480 with a maximum frame rate of 34,700 fps. Two water cooled LED lamps with 400 nm wavelength (ISSI LM2XX-DM-400, Dayton, OH, USA) were used to excite the PSP.

### 3.2. Pressure Sensitive Paint

The pressure sensitive paint (PSP) used in the present study was prepared using the standard formulation of polymer-ceramic pressure sensitive paint (PC-PSP) [3,4]. Details on the sample preparation procedure and its dynamic response characteristics are provided by Pandey and Gregory [18,19], and the paint is commercially available from Innovative Scientific Solutions, Inc., Dayton, OH, USA. The paint is comprised of a large concentration of ceramic particles (TiO${}_{2}$ in this case, with a median particle size of 410 nm), and a small amount of polymer (3% by weight) to serve as a binder. Once thoroughly mixed, the basecoat was airbrushed onto the back wall of the acoustic cavity and left under room conditions to cure. The luminophore used in the present study is platinum tetra (pentafluorophenyl) porphyrin (PtTFPP), a solution of which was oversprayed onto the polymer/ceramic basecoat. The flat frequency response of this formulation of polymer-ceramic PSP is about 7 kHz, defined as the frequency associated with 3-dB attenuation of the signal [19].

### 3.3. Data Acquisition and Processing

A summary of test conditions for the present study is given in Table 1, where the theoretical resonance frequency ($f_{\text{theory}}$) was calculated at a temperature of $22{\;}^{\circ }$C. For the combined multi-frequency mode, the top speaker was used to most efficiently excite the (2,1,0) mode and the side speaker excited the (1,2,0) mode. Each data record had a total of 500 images that were used for subsequent analysis.

**Figure 3:**
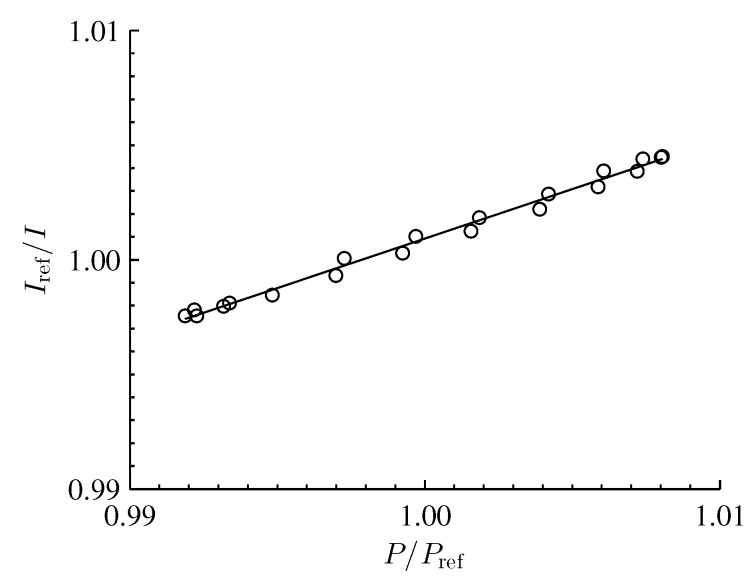
*In situ* calibration of PC-PSP.

The PSP was calibrated via an *in situ* calibration, where phase-averaged data from the pressure transducer were directly compared with an average PSP intensity over a 5×5 pixel region of the paint adjacent to the transducer. Data from both the transducer and the PSP were phase averaged and sampled over 20 phase-averaged bins of the waveform for the (1,1,0) mode. Following this, an intensity ratio, $I_{\mathrm{ref}}/I$, is formed and related to a ratio of the instantaneous pressure to the reference (atmospheric) pressure, $P/P_{\mathrm{ref}}$, by the Stern–Volmer equation,

(18) $$\frac{I_{\mathrm{ref}}}{I}=A+B\frac{P}{P_{\mathrm{ref}}}$$

where *A* and *B* are calibration coefficients for a specific PSP formulation. While the calibration coefficients, *A* and *B*, are dependent upon the local temperature of the fluid and the substrate material, the effects of temperature are assumed to be negligible in this case. The validity of this assumption was handled by Gregory *et al.* [7] and Disotell and Gregory [20] for the exact same PSP formulation, experimental setup, and driving amplitude. The calibration results are shown in Figure 3 with Stern–Volmer coefficients of A=0.57 and B=0.43.

An averaged dark image was subtracted from each of the raw PSP images to reduce dark current noise. The mean of the raw PSP images was used as the reference image, since the mean acoustic pressure field was equal to ambient. The measured calibration (see Figure 3) was applied to the image set to convert intensity ratio to pressure ratio. To further mitigate the effects of noise, a spatial filter was applied to the data using a moving circular window with a radius of 15 pixels; this window size corresponds to approximately 3.1% of the maximum box dimension.

For comparison with DMD results, phase averaging of PSP images was performed using the cyclic acoustic signal information obtained from the reference transducer. The anti-nodal positioning of the transducer enables correlation of the pressure signal with the intensity signal obtained from the top left corner of the image. Image timing information was obtained from the camera shutter signal recorded simultaneously with the transducer signal in the oscilloscope. To perform phase averaging, a sine fit was first applied to the transducer data to obtain phase information of images throughout the acquisition period. Every cycle of the sine curve fit was then divided into 20 bins of $18^{\circ }$ each, and the set of images falling within each bin was averaged over all cycles of the record. The phase bin corresponding to maximum pressure fluctuation is referred to as a $90^{\circ }$ phase, in keeping with the definition of the driving sinusoidal waveform.

## 4. Results and Discussion

The surface pressure distribution in the acoustic resonance box has been previously verified by Gregory *et al.* [7] using phase-locked excitation for the intensity-based method, and by Disotell and Gregory [20] using a single-shot lifetime method. Whereas neither of those approaches provides a continuous time record of pressure, the present data uses high-speed imaging to provide a time-resolved data record that is amenable to advanced analysis. The results presented in the sections to follow are for the three test cases described in Table 1. For mode shape (1,1,0), the results obtained from the analytical solution (Equation (16)) and from phase-averaging are compared with DMD. This serves as a validation for the DMD algorithm. A higher-order mode shape (2,2,0) with a driving frequency of 2595 Hz is presented next. Finally, a comparison of POD and DMD results is presented on multi-frequency excitation, yielding verification and insight that would not be possible with phase-averaging or single-shot techniques.

### 4.1. Mode Shape (1,1,0)

**Figure 4:**
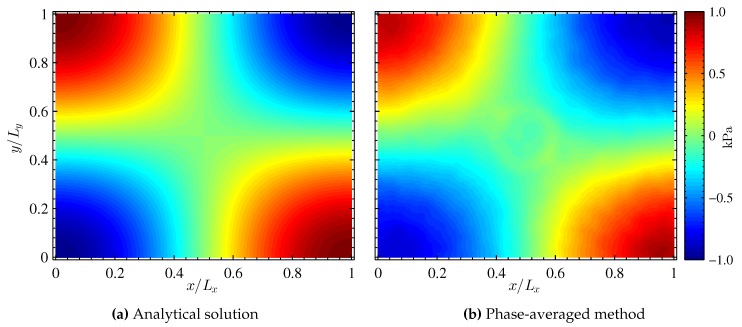
Analytical solution and phase-averaged PSP data for mode shape (1,1,0).

The theoretical pressure field for mode shape (1,1,0) given by Equation (16) is presented in Figure 4a. The mode shape is symmetric about the diagonal of the image, and the nodal lines are mutually orthogonal to one another as predicted by linear acoustic theory [17]. An initial view of the PSP data is provided by the phase-averaged data at $90^{\circ }$ phase shown in Figure 4b. The pressure scale is shown as fluctuations about the mean measured pressure distribution (assumed to be uniformly equal to ambient pressure). Figure 4 shows very good qualitative agreement between theory and measurements. In the phase-averaged result, a circular imprint in the center of the pressure field is seen, which is an artifact from the construction of the box itself. In addition, the node lines indicated in the PSP data are slightly skewed, which is a characteristic of the expected pressure field at the present conditions that are on the verge of the nonlinear acoustic regime.

**Figure 5:**
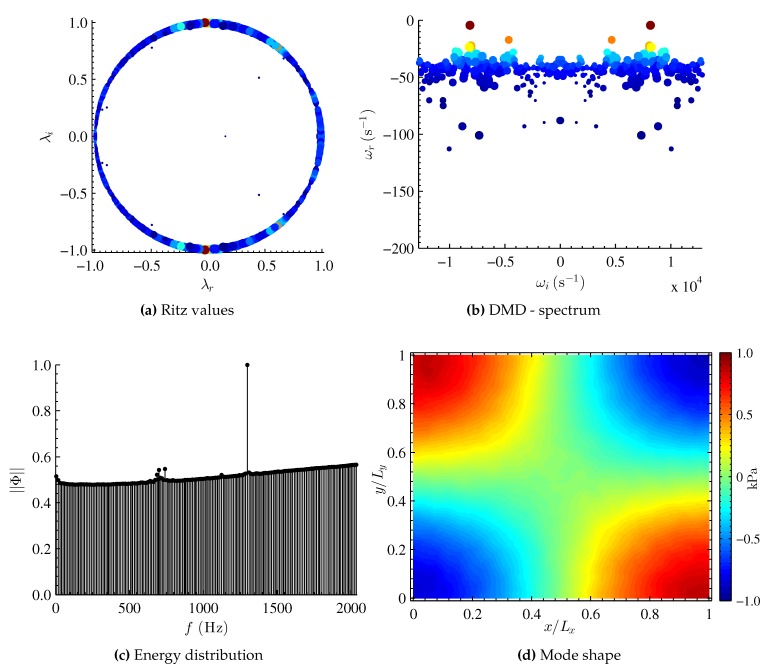
Dynamic mode decomposition (DMD) results for mode shape (1,1,0).

The results of the DMD analysis are presented in Figure 5. The real and imaginary parts of the computed Ritz values ($\lambda_{r}$ and $\lambda_{i}$, respectively) of the approximate full matrix $\tilde{S}$ are shown in Figure 5a. The eigenvalues mostly lie on the unit circle. The color and size of each symbol indicates the amplitude of the eigenvalue in the snapshot data sequence. For mode shape (1,1,0), there is only one dominant flow feature. The eigenvalues corresponding to the dominant mode lie at (-0.028±0.998) in the complex plane. The eigenvalues are also mapped logarithmically (Equation (13)) and are shown in Figure 5b. Again, the size and color of the symbols (mapped eigenvalues) indicate the amplitude of the data sequence. The mapped eigenvalues occur as complex conjugate pairs, and the DMD spectrum is symmetric about $\omega_{i}=0$. The mode of interest lies at ω=8154 s${}^{-1}$ (f=1298 Hz), which is in very good agreement with the theoretical frequency of 1300 Hz. The global energy norm (normalized amplitude *vs.* frequency) for each dynamic mode is plotted in Figure 5c. Sensor noise from the CMOS camera is also picked up in the energy distribution (f∼700-750 Hz). Barring this noise source, the energy distribution shows one energetic structure (dynamic mode) occurring at f=1298 Hz. Thus, the computed resonance frequency is in very good agreement with the theoretical driving frequency of the speaker.

The mode shape corresponding to f=1298 Hz is shown in Figure 5d. The obtained mode shape is in very good agreement with the theoretical and phase-averaged results shown in Figure 4. The present results are also in good agreement with results obtained by the phase-averaged intensity based method [7] and from the single-shot lifetime method [20]. The mode shape is symmetric about the diagonal of the image and the nodal lines are approximately orthogonal to one another. It is worth noting that the circular imprint that was seen in the phase-averaged method (Figure 4b) is not visible in the DMD results. This is due to the fact that the circular imprint is a mean feature (f=0 Hz) and is thus not visible at the dominant structure (f=1298 Hz).

### 4.2. Mode Shape (2,2,0)

**Figure 6:**
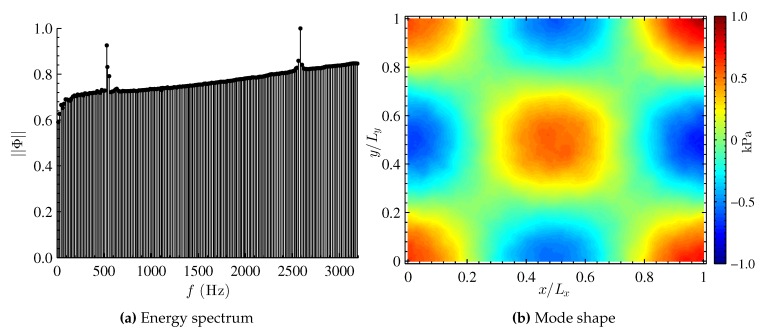
DMD results for mode (2,2,0), with a theoretical frequency of 2594 Hz and a measured frequency of 2584 Hz.

Higher mode shapes are obtained by exciting the PSP at a frequency of 2594 Hz in the acoustic resonance box at 148 dB. The resulting energy distribution from DMD and the corresponding mode shape are shown in Figure 6. Similar to mode shape (1,1,0), the energy distribution shows sensor noise and the dominant mode. During experimentation, it was observed that the frequency of the noise in the spectrum was dependent upon the sampling frequency of the camera. For this higher sample frequency, the noise occurs at a lower range of frequencies between f∼500-550 Hz. The dominant frequency corresponding to mode shape (2,2,0) is at f=2585 Hz. The obtained results from DMD are again in very good agreement with theory for a single dominant flow structure. DMD of the (2,2,0) mode effectively highlights one of the key advantages of PSP, where high spatial resolution can reveal the intricate structure of higher order modes.

### 4.3. Combined Mode Shapes (2,1,0) and (1,2,0)

It has been demonstrated in Section 4.1 that the results of the analytical solution, phase-averaging method, and DMD are in good agreement for the pressure field with single dominant structure. In this section, the effect of simultaneous excitation of the PSP in the acoustic resonance box is analyzed. The resonance box is equipped with two speakers (see Figure 2), where the top speaker is driven at a frequency of 1897 Hz that corresponds to mode shape (2,1,0) and the side speaker is driven at a frequency of 2202 Hz corresponding to a mode shape (1,2,0). The speaker amplitude is adjusted individually such that the pressure with a single speaker is 147 dB. The combined output signal from the pressure transducer mounted in the resonance box resembles a beat frequency. Conventional phase-averaging is *not* possible for this case since a very long time record would be required for sufficient phase averaging at the beat frequency. Thus, the utility of POD and DMD will be demonstrated for extraction of the modes from the time record of pressure.

#### 4.3.1. POD Analysis

**Figure 7:**
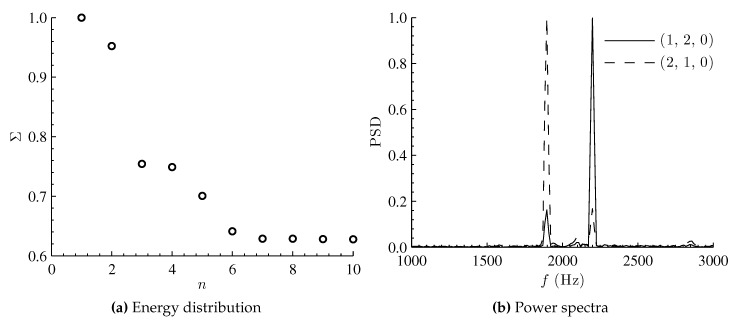
Energy distribution and power spectra of temporal proper orthogonal decomposition (POD) modes for combined modeshapes (2,1,0) and (1,2,0).

The POD algorithm uses the second-order statistics as basis for the decomposition. The decomposition produces a sequence of coherent structures by diagonalizing the spatial correlation tensor and ranks the structures based on their energy content. The singular value decomposition of the combined mode shape snapshot sequence is carried out. The resulting singular values (Σ) are plotted in Figure 7a. As the mode number increases, the magnitude of the normalized singular values rapidly reduces following the first two POD modes. The POD first (n=1) and second (n=2) modes correspond to mode shapes (1,2,0) and (2,1,0), respectively. POD modes n= 3, 4, and 5 are due to sensor noise. The normalized power spectra “of the first two” POD coefficients (‘chronos’) corresponding to modes shapes (1,2,0) and (2,1,0) are shown in Figure 7b. Each curve in the plot has three peaks—the dominant peak, a secondary peak, and a very low-level third peak. The third peak at 2100 Hz has a very low amplitude and is due to the sensor noise from the CCD. The curve corresponding to mode shape (1,2,0) (solid line) has the dominant peak at 2200 Hz. Simultaneously, a secondary peak at 1897 Hz (associated with mode shape (2,1,0)) is also seen in the power spectra of mode (1,2,0). Similarly, for mode shape (2,1,0) (dashed line), the dominant peak is at f=1897 Hz and a secondary peak (associated with mode (1,2,0)) is at 2200 Hz. The occurrence of secondary peaks along with the dominant peaks in each curve is due to the inherent nature of the POD algorithm that relies on modal orthogonality rather than temporal dynamics. The result is that POD cannot completely discriminate between the two modes (frequencies) that are simultaneously present.

#### 4.3.2. DMD Analysis

**Figure 8:**
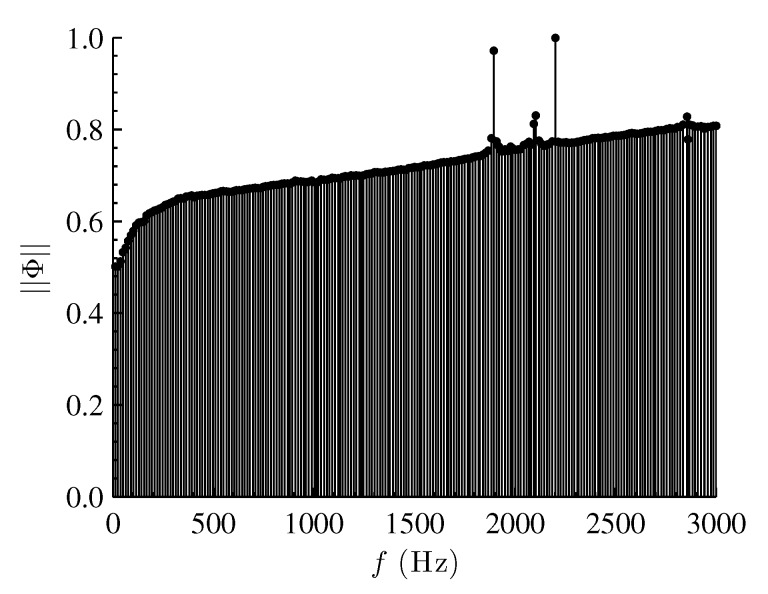
DMD energy spectrum of combined modes (2,1,0) and (1,2,0).

The DMD energy spectrum of the combined modes (2,1,0) and (1,2,0) is plotted in Figure 8. Similar to POD, there are three distinct peaks in the spectrum. Identification of structures is based on the amplitude of the dynamic modes rather than a hierarchical ranking of coherent structures based on their energy content, as in POD. The peak with the lowest amplitude corresponds to the sensor noise from the CCD (f≈2100 Hz). The two dominant peaks of interest are at 1897 and 2200 Hz corresponding to mode shapes (2,1,0) and (1,2,0), respectively. As discussed earlier, the POD algorithm is based on spatial orthogonality, whereas both the temporal (frequency content) and the spatial orthogonality are enforced by the DMD algorithm [15].

#### 4.3.3. Comparison of POD and DMD

**Figure 9:**
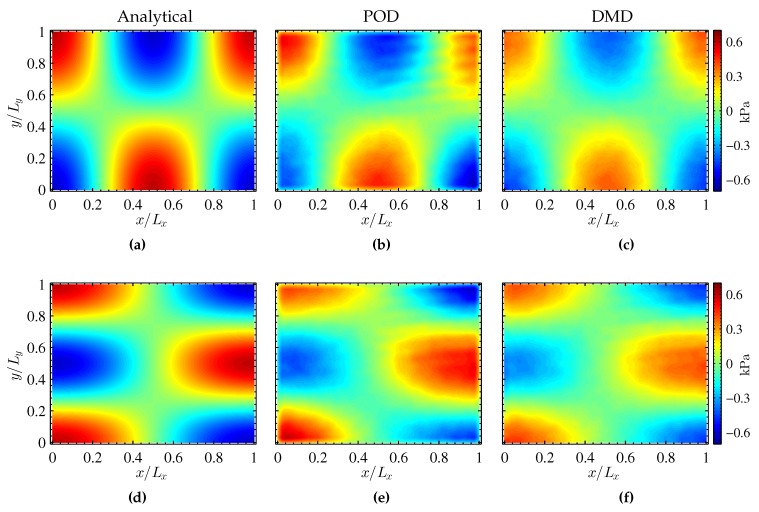
Comparison of decomposed mode shapes (2,1,0) (**Top** row) and (1,2,0) (**Bottom** row).

The mode shapes corresponding to simultaneous excitation of PSP in the resonance box are given in Figure 9. The top and bottom rows of the figure show mode shapes (2,1,0) and (1,2,0), respectively. The mode shapes corresponding to the analytical solution are given in Figure 9a,d. The contours show the pressure distribution and the nodal lines within the resonance box. The results from POD are given in Figure 9b,e. A closer look at the POD results shows that the data is contaminated by noise. The noise occurs in the form of horizontal stripes and is visible near the top right corner ($x/L_{x}=y/L_{y}=0.6∼1$). Since the noise is associated with the CMOS camera sensor, background subtraction does not eliminate the noise. While the spatial orthogonality of the POD algorithm gives two distinct structures, it is not able to eliminate the noise.

The mode shapes from DMD are presented in Figure 9c,f for discrete frequencies of 1897 and 2200 Hz, respectively. Due to the nature of the DMD algorithm used, the temporal orthogonality is enforced along with spatial coherence. At f=1897 Hz, mode shape (2,1,0) forms in the resonance cavity and is clearly seen in Figure 9c. Similarly, at f=2200 Hz, mode shape (1,2,0) is seen in Figure 9f. Upon comparison with POD results, it can been observed that the noise present in POD results is eliminated by DMD. The resulting pressure fields are not contaminated by sensor noise and resemble the analytical solution very closely. Instead of appearing in the dominant modes, the noise appears exclusively in the modes near f≈2100 Hz (see Figure 8).

## 5. Conclusions

The pressure distribution within an acoustic resonance cavity was measured using Fast PSP. Conventional methods for data reduction such as phase-averaging were compared with proper orthogonal decomposition and dynamic mode decomposition. Results from the three methods were found to be in good agreement with one another for single frequency dynamics. While phase-averaging was possible when a single frequency of interest can be identified, POD and DMD were much more effective in cases where multiple frequencies of interest were present. This was clearly seen from the results of the simultaneous excitation of the PSP at two different frequencies. The analysis demonstrates that DMD was more robust than POD in discriminating between the two modes and at eliminating noise from the CMOS sensor. In problems with multiple dominant structures, DMD offers a better choice due to the algorithm’s enforcement of both temporal and spatial orthogonality. Additionally, the size of the data set required for DMD is smaller than phase-averaging, thereby reducing the errors introduced by temperature variations and photodegradation of PSP during the data acquisition process. The results presented here further demonstrate the capabilities of unsteady PSP with improved data reduction methods.

## Figures and Tables

**Table 1:** Summary of test conditions.

Speaker	Mode	$f_{\text{theory}}$ (Hz)	$f_{s}$ (Hz)	Cycles/Record	SPL (dB)

Top	(1,1,0)	1300	5100	127	151
Side	(2,2,0)	2594	8000	162	148
Top & Side	(2,1,0) & (1,2,0)	1897 & 2202	6600	144 & 167	147

## Abbreviations

The following abbreviations are used in this manuscript:

PSP: Pressure Sensitive Paint
PC-PSP: Polymer Ceramic-Pressure Sensitive Paint
DMD: Dynamic Mode Decomposition
POD: Proper Orthogonal Decomposition
SVD: Single Value Decomposition
FFT: Fast Fourier Transform
LED: Light Emitting Diode
CCD: Charge-Coupled Device
CMOS: Complementary Metal-Oxide Semiconductor
